# POEMS syndrome presenting as presumed chronic glomerulonephritis with a 1-year delay in diagnosis: a case report

**DOI:** 10.3389/fmed.2026.1900768

**Published:** 2026-08-27

**Authors:** Li Liu, Mingpeng Li, Fugang Li

**Affiliations:** Department of Nephrology, The People's Hospital of Jianyang City, Chengdu, Sichuan, China

**Keywords:** case report, chronic glomerulonephritis, diagnostic delay, POEMS syndrome, VEGF–vascular endothelial growth factor

## Abstract

**Background:**

Polyneuropathy, Organomegaly, Endocrinopathy, Monoclonal protein, Skin changes (POEMS) syndrome is a rare paraneoplastic disorder driven by an underlying plasma cell dyscrasia. While renal involvement is common, it typically manifests after neurological or dermatological symptoms. Chronic glomerulonephritis-like manifestations as the sole initial feature for a prolonged period are exceptionally rare and can lead to delayed diagnosis.

**Case presentation:**

A 38-year-old woman presented with a 1-year history of foamy urine and a 1-week history of lower limb muscle pain. She had previously been treated with irbesartan for presumed chronic glomerulonephritis. Two months prior to admission, she developed lower limb numbness, followed by progressive muscle pain. Physical examination revealed cervical lymphadenopathy and left-sided cardiomegaly. Laboratory investigations demonstrated proteinuria, mild renal impairment, and a serum monoclonal immunoglobulin A (IgA)-*λ* protein on immunofixation electrophoresis. Nerve conduction studies confirmed severe demyelinating polyneuropathy. Imaging revealed pericardial effusion, splenomegaly, and bilateral renal enlargement. Following multidisciplinary consultation, subsequent investigations revealed markedly elevated serum vascular endothelial growth factor (VEGF) and patchy, slightly high-density shadows in bilateral iliac bones on abdominal computed tomography (CT), which supported the definitive diagnosis of POEMS syndrome. At follow-up, the patient was initiated on lenalidomide and dexamethasone therapy and is currently undergoing autologous stem cell transplantation at a tertiary care center, showing significant symptomatic improvement.

**Conclusion:**

In patients with an unexplained chronic glomerulonephritis-like presentation that is refractory to standard therapy, POEMS syndrome should be considered in the differential diagnosis, even in the absence of typical skin or bone changes. Early serum immunofixation and VEGF assessment are critical for avoiding a delayed diagnosis.

## 1 Introduction

Polyneuropathy, Organomegaly, Endocrinopathy, Monoclonal protein, Skin changes (POEMS) syndrome is a rare paraneoplastic disorder driven by an underlying plasma cell dyscrasia, with an estimated prevalence of less than 1 per 100,000 (1). Its marked clinical heterogeneity frequently results in delayed diagnosis. Renal involvement occurs in approximately 50% of patients, but it usually presents as mild proteinuria or membranoproliferative glomerulonephritis and typically follows or coincides with neurological or dermatological symptoms (2, 3). When kidney disease presents as the sole initial manifestation and persists as the predominant clinical feature for over a year, it may be easily misdiagnosed as primary chronic glomerulonephritis. We report a case of POEMS syndrome presenting as presumed chronic glomerulonephritis with a 1-year delay in diagnosis to raise awareness among nephrologists regarding this atypical presentation.

## 2 Case presentation

### 2.1 Timeline

- 12 months before admission: Onset of foamy urine; urinalysis revealed 2 + proteinuria. The patient was treated with irbesartan for presumed chronic glomerulonephritis, but proteinuria persisted.
- 2 months before admission: Development of bilateral lower limb numbness.
- 1 week before admission: Numbness progressed to muscle pain. Electromyography (EMG) demonstrated demyelinating polyneuropathy.
- Day 0 (20 December 2025): Admission.
- Days 1–3: Serum immunofixation electrophoresis identified an immunoglobulin A (IgA)-*λ* monoclonal protein. Imaging revealed pericardial effusion, splenomegaly, and bilateral renal enlargement.
- Day 15: Serum vascular endothelial growth factor (VEGF) level was markedly elevated at 1009.97 pg./mL. Computed tomography (CT) showed patchy slightly high-density shadows in bilateral iliac bones. Right inguinal lymph node biopsy showed lymphoproliferative lesions. The diagnosis of POEMS syndrome was confirmed.
- Follow-up: The patient received lenalidomide and dexamethasone therapy for 3 months, followed by autologous stem cell transplantation. Post-treatment assessments showed a decrease in VEGF levels to 65 pg./mL, disappearance of the M protein, normalization of renal function, and significant improvement in neurological symptoms.

### 2.2 Patient information

A 38-year-old Han Chinese woman was admitted to our Department of Nephrology with a chief complaint of foamy urine for 1 year and lower limb muscle pain for 1 week.

### 2.3 Clinical history

One year prior to admission, the patient noted persistent foamy urine. Urinalysis at that time showed 2 + proteinuria, leading to a presumptive diagnosis of chronic glomerulonephritis. She was treated with irbesartan and piperazine ferulate tablets; however, her symptoms persisted, and the urine albumin-creatinine ratio (ACR) remained elevated at 256.4 mg/g (reference range: 0–30 mg/g). Two months before admission, she developed numbness in both lower limbs, which she initially ignored. One week before admission, the numbness progressed to severe muscle pain. Electromyography demonstrated multifocal peripheral neuropathy (radiculopathy) involving both sensory and motor fibers in the upper and lower limbs, characterized primarily by demyelination with concomitant axonal involvement.

### 2.4 Past and family history

The patient denied any history of hypertension, diabetes, or autoimmune disease. Her menstrual cycles had been irregular, and she reported no menstrual bleeding for the past 6 months. Her paternal aunt died of “uremia.” No other significant personal or family history was noted.

### 2.5 Physical examination on admission

On admission, vital signs were as follows: temperature 36.5 °C, pulse 96 beats/min, respiratory rate 20 breaths/min, and blood pressure 125/101 mmHg. The patient exhibited a nephrotic facies. Multiple non-tender cervical lymph nodes were palpable. Cardiac examination revealed leftward displacement of the apical beat. The spleen was palpable below the costal margin. No peripheral edema or skin changes were observed.

### 2.6 Investigations on admission

#### 2.6.1 Laboratory findings

Renal function: Urea 9.79 mmol/L (reference range: 2.6–9.5 mmol/L), serum creatinine 103 μmol/L (reference range: 41–73 μmol/L), eGFR (CKD-EPI) 61.93 mL/min/1.73m^2^, uric acid 459 μmol/L (reference range: 155–357 μmol/L).

Urinalysis: Protein (±). Urine ACR 174.89 mg/g (reference range: 0–30 mg/g). Twenty-four-hour urine protein was 420.5 mg/24 h (reference range: 24–141 mg/24 h).

Iron metabolism: Transferrin (TRF) 1.97 g/L (reference range: 2–3.6 g/L), iron (Fe) 8.70 μmol/L (reference range: 7.8–32.2 μmol/L), total iron binding capacity (TIBC) 44.70 μmol/L (reference range: 45–75 μmol/L), transferrin saturation (TS) 19.46% (reference range: 20–55%).

Endocrinology: Thyroid-stimulating hormone (TSH) 5.73 μIU/mL (reference range: 0.72–4.2 μIU/mL).

Immunology: Antinuclear antibody (ANA) negative, antineutrophil cytoplasmic antibody (ANCA) negative, normal complement levels and immunoglobulins. IgA 3.82 g/L (reference range: 1.0–4.2 g/L), IgG 14.60 g/L (reference range: 8.6–17.4 g/L), IgM 1.99 g/L (reference range: 0.5–2.8 g/L), complement C3 1.080 g/L (reference range: 0.7–1.4 g/L), complement C4 0.274 g/L (reference range: 0.1–0.4 g/L).

**Figure 1:**
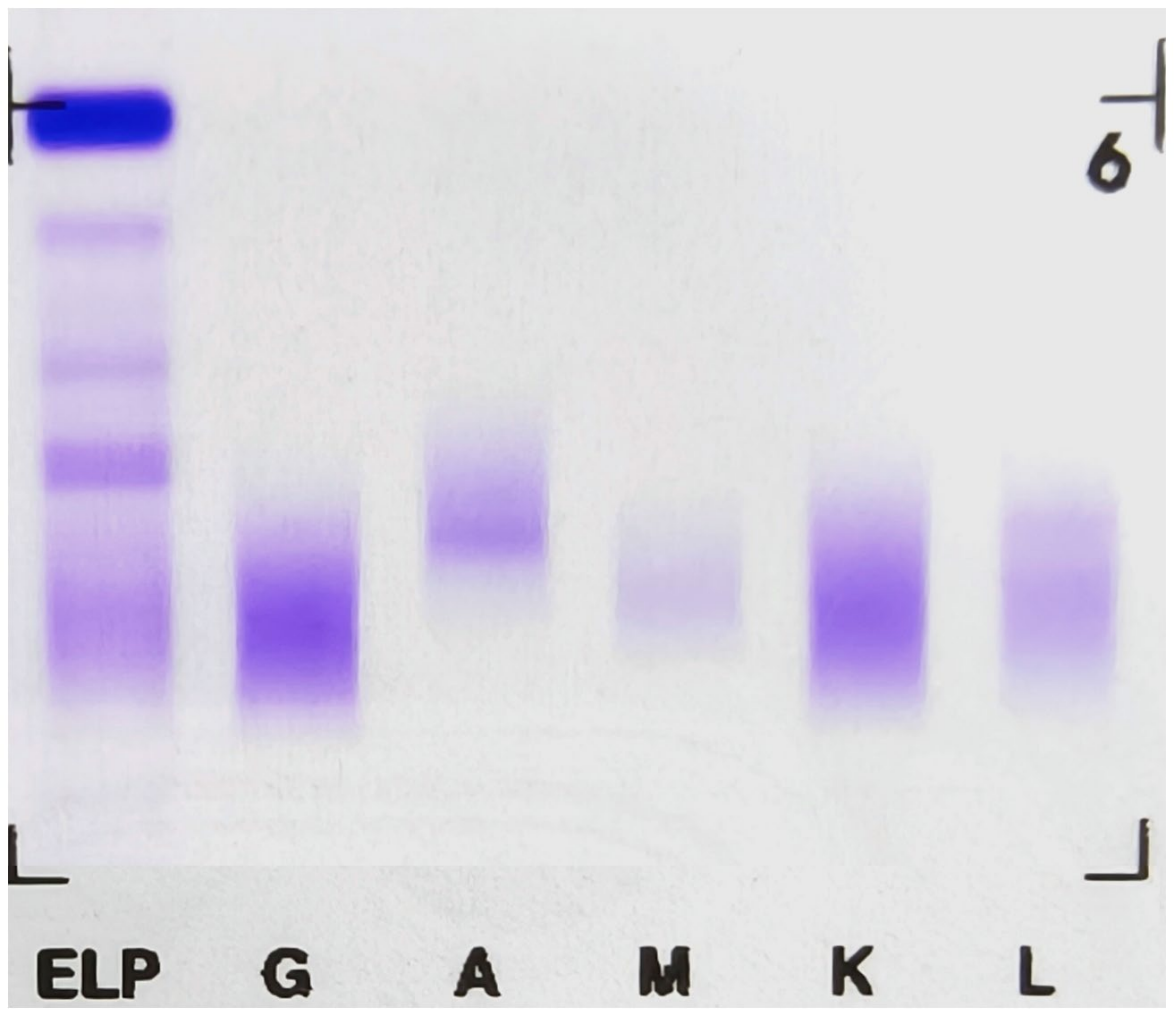
Serum immunofixation electrophoresis showing a weak monoclonal immunoglobulin A (IgA)-*λ* protein (positive). The arrow indicates the monoclonal band in the IgA-λ lane. Other lanes showed no detectable bands.

Serum immunofixation electrophoresis: A weak monoclonal IgA-*λ* protein was detected (positive) (Figure 1).

Other investigations: Complete blood count results were within normal limits, with a platelet count of 283 × 10^9^/L (reference: 100–300 × 10^9^/L), hemoglobin of 149 g/L (reference: 115–150 g/L), and hematocrit of 43% (reference: 35–45%). Additionally, C-reactive protein, total bilirubin, direct bilirubin, total protein, albumin, alanine aminotransferase (ALT), aspartate aminotransferase (AST), and fasting blood glucose were all within normal limits.

A summary of the main abnormal laboratory findings is presented in Table 1.

**Table 1:** Summary of main abnormal laboratory findings.

Category	Parameter	Result	Reference range
Renal function	Serum creatinine	103 μmol/L	41–73
	eGFR (CKD-EPI)	61.93 mL/min/1.73 m^2^	>90
	Uric acid	459 μmol/L	155–357
	Urine ACR	174.89 mg/g	0–30
	24 h urine protein	420.5 mg/24 h	24–141
Endocrine	TSH	5.73 μIU/mL	0.72–4.2
Monoclonal protein	Serum immunofixation	IgA-λ monoclonal protein (weakly positive)	Negative
	Serum free light chain κ	67 mg/L	6.70–22.40
	Serum free light chain λ	109 mg/L	8.30–27.00
	Urine κ light chain	35.6 mg/L	0–7.75
	Urine λ light chain	18 mg/L	0–4.25
Other	VEGF	1009.97 pg./mL	0–142

ACR, albumin-to-creatinine ratio; CKD-EPI, Chronic Kidney Disease Epidemiology Collaboration; eGFR, estimated glomerular filtration rate; IgA, immunoglobulin A; TSH, thyroid-stimulating hormone; VEGF, vascular endothelial growth factor.

#### 2.6.2 Imaging and other studies

**Figure 2:**
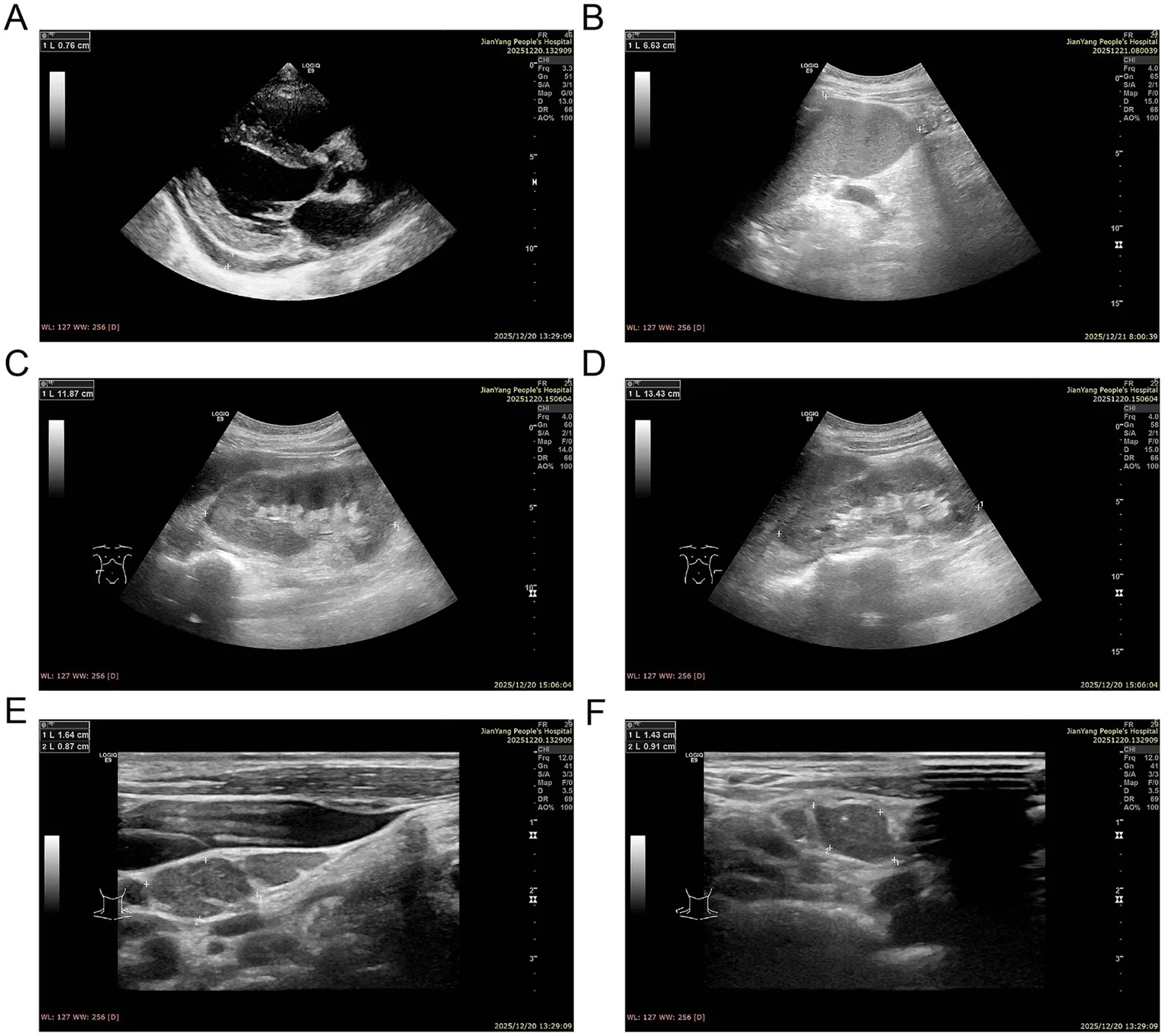
Ultrasound findings. **(A)** Echocardiography demonstrating pericardial effusion. **(B)** Abdominal ultrasound showing splenomegaly. **(C,D)** Renal ultrasound revealing bilateral kidney enlargement. **(E,F)** Cervical ultrasound showing bilateral enlarged cervical lymph nodes with cortical thickening.

Echocardiography revealed mild thickening of the interventricular septum and left ventricular posterior wall, with a pericardial effusion (maximum depth, 8 mm) (Figure 2A). No valvular abnormalities or intracardiac shunts were detected.

Abdominal ultrasound demonstrated splenomegaly (intercostal thickness 5.6 cm, subcostal length 6.6 cm) (Figure 2B) and bilateral renal enlargement (right kidney 13.4 × 6.5 × 5.7 cm, left kidney 13.4 × 6.2 × 4.9 cm) (Figures 2C,D). The liver, gallbladder, and pancreas were unremarkable.

Cervical ultrasound revealed bilateral enlarged cervical lymph nodes with cortical thickening, as well as a mixed-echogenicity area in the right supraclavicular fossa (Figures 2E,F).

**Figure 3:**
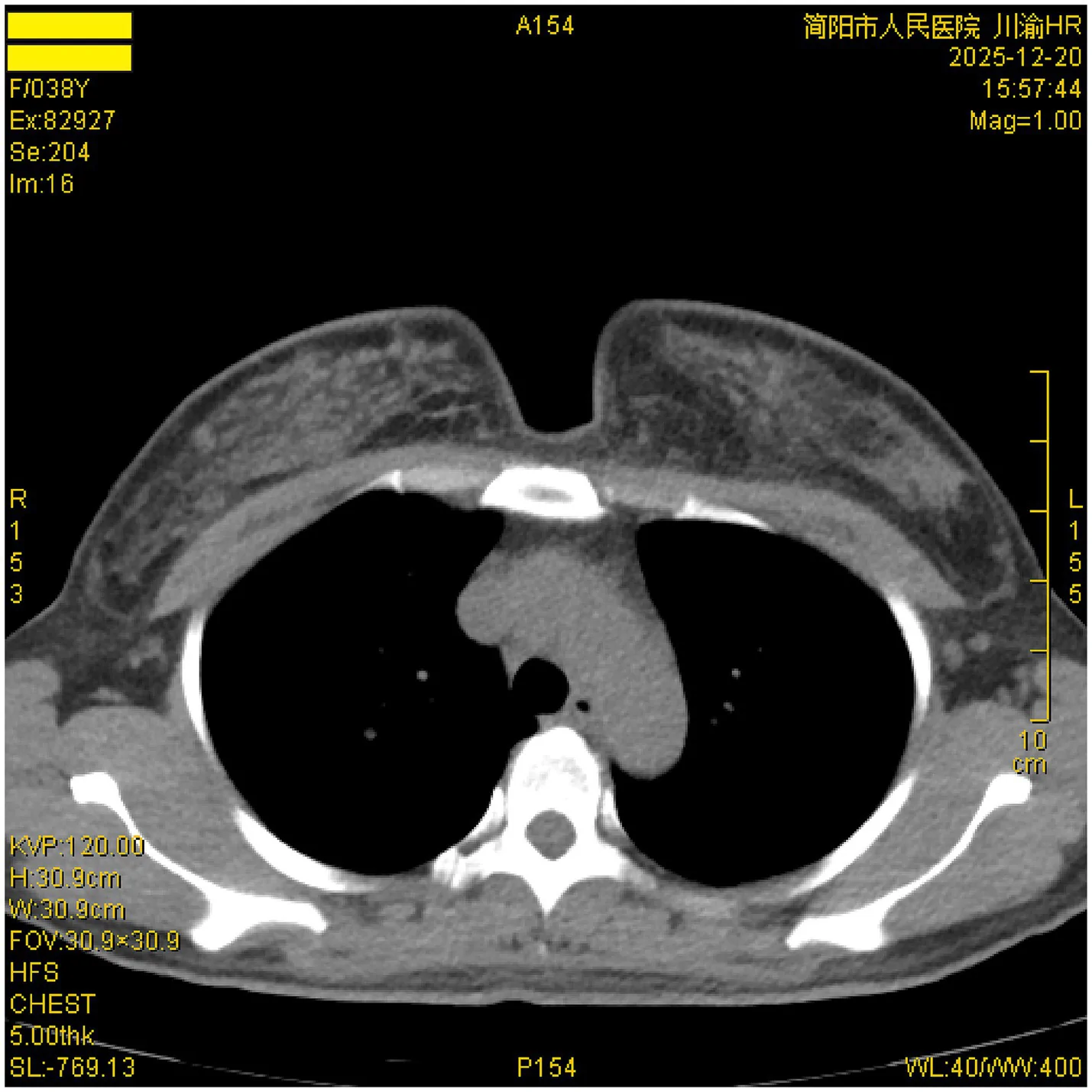
Chest computed tomography (CT) scan. CT image demonstrating pericardial effusion.

Chest computed tomography (CT) confirmed pericardial effusion (maximum thickness 2.4 cm) and marked splenomegaly (Figure 3). No mediastinal lymphadenopathy or pleural effusion was observed. The lung parenchyma showed only minimal inflammatory changes in the right middle lobe and left lingula.

Lumbar spine magnetic resonance imaging revealed preserved physiological curvature of the lumbar spine. A concave deformity with marginal sclerosis was observed at the superior endplate of the L3 vertebral body, consistent with a Schmorl’s node. The remaining vertebral bodies and intervertebral discs showed no significant abnormalities. The spinal canal, dural sac, and conus medullaris were unremarkable. Subcutaneous soft tissue swelling with patchy STIR hyperintensity was noted in the lumbar region, suggestive of lumbar fasciitis.

Nerve conduction studies confirmed severe demyelinating sensorimotor polyneuropathy.

### 2.7 Final diagnosis

Upon admission, the initial working diagnoses included presumptive chronic glomerulonephritis, cervical lymphadenopathy, and polyneuropathy of unknown etiology. The patient was initially managed with irbesartan. Given the multisystem involvement of the kidneys, nervous system, lymph nodes, pericardium, spleen, and endocrine system, coupled with negative autoantibody testing, the attending physicians suspected POEMS syndrome, Castleman disease, or IgG4-related disease. A multidisciplinary team comprising specialists in neurology, rheumatology, cardiology, and plastic surgery recommended further investigations, including serum immunofixation electrophoresis, vascular endothelial growth factor (VEGF) measurement, lymph node biopsy, bone marrow aspiration, and renal biopsy.

Following further investigation, the patient was found to have a markedly elevated serum VEGF level of 1009.97 pg./mL (reference range: 0–142 pg./mL). Serum free light chain analysis revealed elevated *κ* and *λ* at 67 mg/L (reference range: 6.70–22.40 mg/L) and 109 mg/L (reference range: 8.30–27.00 mg/L), respectively, with a κ/λ ratio of 0.61, which was within the normal range (reference range: 0.31–1.56). Urine immunofixation demonstrated elevated free light chains as well, with urine κ light chain at 35.6 mg/L (reference range: 0–7.75 mg/L) and λ light chain at 18 mg/L (reference range: 0–4.25 mg/L), yielding a urine κ/λ ratio of 1.98 (reference range: 0.75–4.50) (Table 1). Abdominal CT imaging revealed splenomegaly, bilateral renal enlargement, and patchy, slightly high-density shadows in the bilateral iliac bones. A right inguinal lymph node biopsy revealed lymphoproliferative lesions. Neurological examination demonstrated reduced distal superficial sensation and diminished or absent tendon reflexes in the lower limbs. However, the patient refused further examinations, including renal biopsy and bone marrow aspiration.

According to the current criteria (1) the final diagnosis of POEMS syndrome was confirmed, as the patient fulfilled both mandatory criteria, at least one major criterion, and two minor criteria. Table 2 summarizes the application of these criteria to our case.

**Table 2:** Diagnostic criteria for POEMS syndrome applied to this case.

Criteria	Requirement	This case	Met
Mandatory (both required)	Polyneuropathy (typically demyelinating)	EMG-confirmed demyelinating polyneuropathy	√
	Monoclonal plasma cell-proliferative disorder (almost always λ)	Serum immunofixation: IgA-λ (+)	√
Major (one required)	Castleman disease	Lymph node biopsy: lymphoproliferative lesions (negative for Castleman)	×
	Sclerotic bone lesions	CT: patchy high-density shadows in bilateral iliac bones	⍻
	VEGF elevation	VEGF 1009.97 pg./mL (reference <142)	√
Minor (one required)	Organomegaly (splenomegaly, hepatomegaly, or lymphadenopathy)	Splenomegaly, lymphadenopathy, bilateral renal enlargement	√
	Extravascular volume overload (edema, pleural effusion, or ascites)	Pericardial effusion	√
	Endocrinopathy (adrenal, thyroid, pituitary, gonadal, parathyroid, pancreatic)	Elevated TSH	⍻
	Skin changes (hyperpigmentation, hypertrichosis, glomeruloid hemangiomata, plethora, acrocyanosis, flushing, white nails)	None	×
	Papilledema	Not examined	?
	Thrombocytosis/erythrocytosis	None	×

CT, computed tomography; EMG, electromyography; IgA, immunoglobulin A; TSH, thyroid-stimulating hormone; VEGF, vascular endothelial growth factor.

### 2.8 Follow-up

The patient received lenalidomide and dexamethasone for 3 months, followed by autologous stem cell transplantation (ASCT). Serial follow-up data were obtained:

VEGF: 1009.97 pg./mL (baseline) → 126 pg./mL (after 3 months of lenalidomide/dexamethasone) → 65 pg./mL (1 month post-ASCT).

Immunofixation electrophoresis: IgA-*λ* positive (baseline and at 3 months) → negative (1 month post-ASCT).

Renal function: Creatinine decreased to 84 μmol/L, and abdominal ultrasound showed resolution of bilateral kidney enlargement.

Neurological symptoms: Lower limb pain and numbness improved significantly. The patient can now walk independently, squat, and lift her legs, though she is still unable to stand on tiptoes, jump, or run.

Other systemic findings: No new symptoms reported.

## 3 Discussion

### 3.1 Multisystem involvement as a cohesive clinical picture of POEMS syndrome

POEMS syndrome is fundamentally a multisystem disorder, and the concurrent involvement of seemingly unrelated organs in a single patient is not coincidental but reflects a unified underlying pathogenesis. Our patient exhibited involvement of four distinct systems that, when viewed collectively, form a cohesive picture characteristic of POEMS syndrome. Renal involvement was the earliest manifestation, presenting as proteinuria, mild renal impairment, and bilateral renal enlargement. Although renal involvement is not a diagnostic criterion for POEMS syndrome, it is observed clinically in a subset of patients, typically manifesting as mild proteinuria (usually <1 g/day) and/or preserved or mildly impaired renal function. Renal biopsy can reveal membranoproliferative-like changes with evidence of endothelial injury and negative immunofluorescence findings, which may help distinguish it from primary membranoproliferative glomerulonephritis (1, 4, 5). Lymphoreticular involvement was evident from marked splenomegaly and extensive lymphadenopathy involving cervical, axillary, and abdominal regions. Organomegaly, including hepatomegaly, splenomegaly, and/or lymphadenopathy, occurs in approximately 45–85% of patients and represents one of the minor diagnostic criteria (1). Cardiovascular involvement was demonstrated by pericardial effusion on both echocardiography and CT. Pericardial and pleural effusions are well-recognized manifestations of POEMS, associated with VEGF-induced vascular permeability and extravascular fluid accumulation; cardiac complications, while relatively rare, can include pericardial effusions, cardiomyopathy, and heart failure (1). The patient exhibited an elevated TSH level, indicating thyroid dysfunction. Endocrinopathy is common in POEMS syndrome, occurring in 67–84% of patients, with hypogonadism being the most frequent pattern, followed by adrenal and thyroid dysfunction (1). The unifying mechanism involves cytokine imbalance driven by the monoclonal plasma cell clone, with VEGF overproduction playing a central role in mediating vascular permeability, endothelial dysfunction, and tissue edema, thereby contributing to serous effusions, organ enlargement, endoneurial edema, and endothelial injury (1, 6). The markedly elevated VEGF level in our patient provides a mechanistic link between these disparate findings, and its subsequent decline following treatment supports VEGF as a key biomarker of disease activity. Thus, the multisystem involvement in this case is not a random assemblage of unrelated pathologies but represents a coherent presentation that is fully consistent with the known clinical spectrum of POEMS syndrome.

### 3.2 Diagnostic pitfalls and key lessons

The most striking feature of this case is that chronic glomerulonephritis-like renal manifestations were the sole clinical presentation before any neurological symptoms emerged. Renal involvement in POEMS syndrome typically occurs after or concurrently with neuropathy and is often accompanied by characteristic cutaneous manifestations, such as erythema, hyperpigmentation, and acrocyanosis (1, 6–8). In contrast, our patient had initially exhibited none of these cutaneous findings. Such a “renal-first, early-phase renal-only, and prolonged” presentation of POEMS syndrome is exceptionally rare and directly contributed to delayed diagnosis.

Another unusual feature is the weakly positive IgA-*λ* monoclonal protein. The monoclonal protein in POEMS syndrome is almost exclusively of the λ light chain type, accounting for over 95% of cases, while the heavy chain distribution varies across cohorts. For instance, a multicenter analysis reported IgG and IgA subtypes in 48 and 47% of cases, respectively, whereas other series have described IgG predominance, reaching up to 72%, with IgA being less common at approximately 18% (1, 9). A weakly positive result can be missed by routine serum protein electrophoresis and requires immunofixation for detection. This underscores the importance of directly ordering immunofixation when POEMS is suspected.

The initial misdiagnosis as chronic glomerulonephritis highlights a critical diagnostic pitfall: POEMS syndrome can masquerade as primary renal disease during its early phase. The patient’s nephrotic facies, proteinuria, and mild renal impairment naturally led clinicians to initially focus the diagnostic workup on glomerular disease. However, the subsequent emergence of demyelinating polyneuropathy, organomegaly, and endocrinopathy should have triggered a shift toward a systemic plasma cell disorder. According to the current diagnostic criteria, POEMS syndrome requires the mandatory presence of polyneuropathy and a monoclonal plasma cell proliferative disorder, plus at least one major criterion and one minor criterion (10). In this case, the diagnosis was ultimately confirmed by the combination of polyneuropathy, monoclonal IgA-*λ* protein, markedly elevated VEGF, sclerotic bone lesions, organomegaly, and endocrinopathy. The markedly elevated VEGF level served as a pivotal diagnostic clue, aligning with the established role of VEGF as a sensitive and specific biomarker for POEMS syndrome (1, 7). Notably, VEGF elevation may be influenced by serum ferritin levels (11). Therefore, hypoferritinemia should be excluded during the diagnostic process. Furthermore, since osteosclerotic lesions are present in approximately 95% of patients with POEMS syndrome, imaging modalities such as CT body bone windows and/or FDG-PET/CT are recommended for detecting sclerotic bone lesions in suspected cases (1, 12).

### 3.3 Differential diagnosis: POEMS syndrome vs. AL amyloidosis

Although the patient’s clinical presentation fulfills the diagnostic criteria for POEMS syndrome, AL amyloidosis represents a critical differential diagnosis. Both conditions can present with a similar constellation of renal involvement, cardiac thickening or pericardial effusion, and peripheral neuropathy in the presence of a monoclonal protein (13). Furthermore, while some cases of POEMS syndrome are associated with concurrent AL amyloidosis, the current evidence supports a primary diagnosis of POEMS syndrome in this case for several reasons. First, the pattern of peripheral neuropathy differed significantly. The patient’s nerve conduction studies demonstrated a predominantly demyelinating polyneuropathy with secondary axonal involvement, and she lacked autonomic symptoms such as orthostatic hypotension. In contrast, peripheral neuropathy associated with AL amyloidosis is predominantly axonal, often accompanied by secondary demyelinating changes; moreover, autonomic dysfunction is highly prevalent, affecting up to 65–94% of patients (14–16). Second, the patient’s serum free light chain (sFLC) ratio was normal. In AL amyloidosis, an abnormal sFLC ratio alone detects monoclonality in 79.5% of cases, while the combination of serum and urine immunofixation electrophoresis (IFE) with the sFLC ratio increases detection sensitivity to 89.8% (13). The absence of a skewed sFLC ratio in our patient makes AL amyloidosis less likely. Third, VEGF levels were markedly elevated and showed a dynamic response to treatment. This pattern of VEGF elevation and its correlation with therapeutic response are characteristic hallmarks of POEMS syndrome, whereas VEGF is not typically elevated in AL amyloidosis (7). Unfortunately, the patient declined further tissue biopsy (renal, nerve, or subcutaneous fat pad) for direct histopathological confirmation. Consequently, AL amyloidosis cannot be entirely excluded, which constitutes a limitation of this case report. However, based on the aforementioned clinical, electrodiagnostic, and biochemical evidence, the diagnosis of POEMS syndrome is well-supported.

### 3.4 Implications for clinical practice

This case reinforces several key lessons for clinical practice:

Early VEGF testing should be incorporated into the initial workup for patients being evaluated for acquired demyelinating neuropathy, especially when there is any hint of monoclonal gammopathy or systemic involvement (1, 7).

Comprehensive monoclonal gammopathy screening should include both serum and urine immunofixation, protein electrophoresis, and light chain quantification, as the monoclonal spike in POEMS syndrome is often subtle and may be missed on electrophoresis alone (1).

Multisystem evaluation is essential. The presence of organomegaly, endocrinopathy, or skin changes should prompt a thorough search for POEMS syndrome, even in the absence of overt neuropathy at presentation (12).

Renal biopsy findings in POEMS syndrome are often nonspecific and may resemble thrombotic microangiopathy or other glomerular diseases, making them unreliable for confirming the diagnosis in isolation (17).

### 3.5 Limitations

We acknowledge several limitations in this case report:

Renal biopsy was not performed, so histopathological confirmation of the renal involvement is lacking.

The patient refused further tissue biopsy (renal, nerve, or fat pad) to directly exclude AL amyloidosis.

Whole-body CT with dedicated bone windows was not performed. A dedicated bone window study would have provided a more comprehensive evaluation.

Comprehensive endocrine workup for amenorrhea (serum prolactin, FSH, LH, and estradiol) was not performed. While the presentation aligns with POEMS-associated hypogonadism, alternative causes such as perimenopause or hyperprolactinemia remain possible.

The follow-up duration was limited; long-term outcomes remain to be observed.

## 4 Conclusion

In conclusion, this case illustrates that POEMS syndrome can present with a prolonged, renal-predominant early phase before the classic neurological and systemic features emerge. The combination of a weak IgA-*λ* monoclonal protein, markedly elevated VEGF, sclerotic bone lesions, organomegaly, and endocrinopathy ultimately led to the correct diagnosis. Clinicians should maintain a high index of suspicion for POEMS syndrome in patients with unexplained proteinuria or renal impairment, particularly when accompanied by subtle systemic signs or when a monoclonal gammopathy is detected. Early recognition and appropriate testing are crucial to avoid delayed diagnosis and ensure timely initiation of targeted therapy.

## Data Availability

The raw data supporting the conclusions of this article will be made available by the authors, without undue reservation.
